# Clinical features, electroencephalogram, and biomarkers in pediatric sepsis-associated encephalopathy

**DOI:** 10.1038/s41598-022-14853-z

**Published:** 2022-06-23

**Authors:** Bruno Espírito Santo de Araújo, Rosiane da Silva Fontana, Maria Clara de Magalhães-Barbosa, Fernanda Lima-Setta, Vitor Barreto Paravidino, Paula Marins Riveiro, Lucas Berbert Pulcheri, Margarida dos Santos Salú, Mariana Barros Genuíno-Oliveira, Jaqueline Rodrigues Robaina, Antonio José Ledo Alves da Cunha, Fernanda Ferreira Cruz, Patricia Rieken Macedo Rocco, Fernando Augusto Bozza, Hugo Caire de Castro-Faria-Neto, Arnaldo Prata-Barbosa

**Affiliations:** 1 Department of Pediatrics, D’Or Institute for Research & Education (IDOR), Rio de Janeiro, RJ 22281-100 Brazil; 2 Department of Neurology, State Institute of the Brain Paulo Niemeyer, Rio de Janeiro, RJ 20230-031 Brazil; 3 Department of Epidemiology, Institute of Social Medicine, State University of Rio de Janeiro (UERJ), Rio de Janeiro, RJ 20550-013 Brazil; 4 Department of Physical Education and Sports, Naval Academy, Brazilian Navy, Rio de Janeiro, RJ 20021-010 Brazil; 5 Pediatric Intensive Care Unit, Hospital Caxias D’Or, Duque de Caxias, RJ 25071-181 Brazil; 6 Pediatric Intensive Care Unit, Hospital Rios D’Or, Rio de Janeiro, RJ 22745-005 Brazil; 7 Instituto de Puericultura e Pediatria Martagão Gesteira, Federal University of Rio de Janeiro (UFRJ), Rio de Janeiro, RJ 21941-912 Brazil; 8 Laboratory of Pulmonary Investigation, Carlos Chagas Filho Institute of Biophysics, Federal University of Rio de Janeiro (UFRJ), Rio de Janeiro, RJ 21941-902 Brazil; 9 Rio de Janeiro Network on Neuroinflammation, Carlos Chagas Filho Foundation for Supporting Research, State of Rio de Janeiro (FAPERJ), Rio de Janeiro, RJ Brazil; 10 Immunopharmacology Laboratory, Oswaldo Cruz Institute (FIOCRUZ), Rio de Janeiro, RJ 21040-900 Brazil; 11 Department of Intensive Care Medicine, D’Or Institute for Research & Education (IDOR), Rio de Janeiro, RJ 22281-100 Brazil

**Keywords:** Biomarkers, Diseases, Neurology, Signs and symptoms

## Abstract

To date, no specific diagnostic criteria for sepsis-associated encephalopathy (SAE) have been established. We studied 33 pediatric patients with sepsis prospectively and evaluated the level of consciousness, the presence of delirium, electroencephalographic (EEG) findings, and plasma levels of neuron-specific enolase and S100-calcium-binding protein-B. A presumptive diagnosis of SAE was primarily considered in the presence of a decreased level of consciousness and/or delirium (clinical criteria), but specific EEG abnormalities were also considered (EEG criteria). The time course of the biomarkers was compared between groups with and without clinical or EEG criteria. The Functional Status Scale (FSS) was assessed at admission, discharge, and 3–6 months post-discharge. Clinical criteria were identified in 75.8% of patients, EEG criteria in 26.9%, both in 23.1%, and none in 23.1%. Biomarkers did not differ between groups. Three patients had an abnormal FSS at discharge, but no one on follow-up. A definitive diagnostic pattern for SAE remained unclear. Clinical criteria should be the basis for diagnosis, but sedation may be a significant confounder, also affecting EEG interpretation. The role of biomarkers requires a better definition. The diagnosis of SAE in pediatric patients remains a major challenge. New consensual diagnostic definitions and mainly prognostic studies are needed.

## Introduction

Sepsis is an important cause of admission to the Pediatric Intensive Care Unit (PICU). Despite the “Surviving Sepsis Campaigns”, mortality rates vary from 4 to 50% depending on several risk factors, including comorbidities and local care structure^1^. Sepsis-associated encephalopathy (SAE) has been defined as a diffuse brain dysfunction not caused by a specific central nervous system infection, and clinically manifested as a disturbance of consciousness, with changes in perception and delirium characteristics, such as inattention, irritability, varying degrees of coma (mild to deep), and more rarely convulsions, tremors, asterixis, or myoclonus^2–6^. Other causes of encephalopathy need to be ruled out, especially structural abnormalities in the central nervous system, like tumors or stroke, and also metabolic encephalopathies, including hepatic and renal^2,4,5,7^.

To date, no consensus on specific clinical, laboratorial, or imaging markers have been described for SAE diagnosis. The increase in Neuron-Specific Enolase (NSE) and S100 calcium-binding protein B (S100B) has been correlated with the clinical picture of delirium associated with sepsis both in adult^8–10^ and pediatric studies^11–13^ The electroencephalogram (EEG) may show theta, delta, triphasic, or burst suppression waveforms, as well as activities compatible with abnormal electrical discharges^2,6,14–16^. Magnetic resonance imaging (MRI) has shown several abnormalities in SAE, such as decreased brain volume, leukomalacia, and areas of ischemia, which has been correlated with the degree of brain injury^17,18^. There is no specific treatment for SAE, and the prognosis is uncertain. The only pediatric study showed a high prevalence of neurodevelopmental and behavioral changes^19^, and long-term cognitive impairment and lower health-related quality of life were reported in adult studies^2,20^. In the current study, clinical and electroencephalographic criteria, usually associated with SAE diagnosis, were evaluated in pediatric patients with sepsis. Moreover, the longitudinal course of the NSE and S100B biomarkers was described. The outcome was assessed by the FSS three and six months after discharge.

## Results

Ninety-eight patients were eligible for the study. Sixty-five were excluded: 21 had proven central nervous system infection (encephalitis or meningitis), 18 provided informed consent only after 72 h of sepsis diagnosis, and 26 refused to participate. No other cause of exclusion was observed. Therefore, 33 patients were included (Supplementary Fig. S1 online). None had complications that could result in their exclusion after the study started. The demographic, epidemiological, and clinical characteristics of these patients are shown in Table 1 and Table S1. The median time from the diagnosis of sepsis to admission to the PICU and inclusion in the study was 1h25min (IQR 00h15-03h40), ranging from 0 to 8h12min (Table S1).

**Table 1:** Demographics, epidemiological, and clinical features of patients with sepsis, evaluated for possible diagnosis of sepsis-associated encephalopathy.

Characteristic	Total, no. (%)
Total	33 (100)
Age, years, median (IQR)	1 (1–2)
Infants (< 12 m)	8 (24.2)
Toddler (≥ 12 m, < 3 years)	20 (60.6)
Preschool (≥ 3 y, < 5 years)	1 (3.0)
Grade-schooler (≥ 5 years, < 12 years)	3 (9.1)
Teen (≥ 12 years, < 18 years)	1 (3.0)
**Sex, n (%)**	
Male	12 (36.4)
Female	21 (63.6)
**Race, n (%)**	
White	17 (51.5)
Black	16 (48.5)
Bodyweight, kg, median (IQR)	11 (9–13.5)
**Type of admission**	
Clinical	31 (100)
Surgical	–
**Source of infection, n (%)**	
Respiratory (pneumonia)	26 (78.8)
Dermatology (cellulitis)	3 (9.1)
Gastrointestinal (gastroenteritis)	2 (6.1)
Urinary	2 (6.1)
Comorbidities, n (%)	7 (21.2)
Asthma	5 (15.2)
Congenital heart defect	1 (3.0)
Mucocutaneous disease	1 (3.0)
**Sepsis diagnosis criteria**^**a**^** n (%)**	
Heart rate (tachycardia)	33 (100)
Respiratory rate (tachypnea)	33 (100)
Temperature (fever)	25 (75.8)
Increased leucocyte number	27 (81.8)
Organ dysfunction at diagnosis^a^ n (%)	33 (100)
Cardiovascular	33 (100)
Respiratory	25 (75.8)
Neurological^b^	4 (12.1)
**PELOD 2 (organ dysfunction)**	
Admission score, median (IQR)	3 (2–5)
No. of organ dysfunctions, median (IQR)	1 (1–2)
PIM3, median (IQR), % death probability	4.6 (1.5–6.0)
Length of stay in PICU, median (IQR)	16 (11–23)
**Outcome**	
Discharge	32 (97)
Death	1 (3)

*IQR* interquartile range, *PELOD 2 *Pediatric Logistic Dysfunction 2, *PIM3 *Pediatric Index of Mortality 3.

^a^Goldstein et al.^35^.

^b^The international consensus criteria for neurological dysfunction consider a Glasgow Coma Score ≤ 11, different from the one adopted in this study (< 15) to diagnose sepsis-associated encephalopathy.

### SAE diagnosis

Table 2 shows the scores for the level of consciousness and delirium, the conclusion of the EEG analysis, and the worst levels of the studied biomarkers on the days they were detected. Hatched cells represent abnormal results for clinical and EEG criteria, suggestive of SAE. Biomarker levels above the reference range are highlighted in bold but were not used for SAE diagnosis. Glasgow Coma Scale (GCS) was not performed in five patients as they were already intubated. Likewise, in 14 patients, delirium evaluated by the Cornell Assessment of Pediatric Delirium (CAPD) scale was not assessed due to heavy sedation, and seven patients did not undergo EEG for logistical reasons.

**Table 2:** Clinical scales and electroencephalographic results used for the presumptive diagnosis of SAE and the worst levels of biomarkers on the days they were detected.

Patient no.^a^	GCS	FOUR scale^b^			CAPD			EEG sugg. of SAE^c^	Biomarkers (pg/mL)^d^—worst levels			
		D1	D2	D3	D1	D2	D3		NSE	Day	S100B	Day
*2*	15	10	13	12	–	*9*	*11*	No	3055.39	D6	259.35	D6
3*	*14*	16	16	16	8	2	0	*Yes*	3032.34	D6	594.91	D2
*4*	15	16	*8*	5	7	–	–	No	4208.69	D6	723.02	D4
*5*	*5*	0	2	2	–	–	–	No	2955.95	D4	278.26	D2
*6*	–	8	10	9	–	–	–	*Yes*	3938.71	D3	349.89	D5
7	15	9	10	2	–	–	–	No	**6683.28**	D2	512.70	D4
*9*	–	1	1	10	–	–	*9*	No	3895.97	D1	**993.86**	D4
10	–	5	7	9	–	–	–	No	4172.45	D2	533.26	D2
*11*	*12*	8	8	8	–	–	–	No	4751.79	D7	474.43	D2
12*	–	11	13	15	*19*	*19*	8	*Yes*	5005.95	D5	619.45	D7
13*	*13*	*14*	*14*	16	8	3	6	*Yes*	4346.12	D4	**1.091.08**	D5
14*	–	8	–	10	*20*	–	*23*	*Yes*	3985.82	D3	622.57	D2
15*	–	6	8	11	–	–	*21*	*Yes*	2806.61	D2	701.74	D3
17	15	–	5	10	–	–	–	No	**10,015.06**	D6	294.28	D5
*19*	15	9	8	6	–	–	*17*	No	4297.90	D3	**1.200.16**	D3
*20*	*14*	16	16	16	4	3	*9*	No	2919.26	D6	314.03	D6
*23*	*10*	5	5	5	–	–	–	No	3050.43	D7	551.30	D6
*24*	15	10	9	13	–	19	8	No	**5710.61**	D7	236.18	D7
*25*	*12*	11	9	10	3	–	–	No	2099.66	D5	561.05	D3
*26*	15	*14*	*14*	*15*	*11*	*9*	*9*	No	1239.07	D5	**961.42**	D5
27	15	2	2	5	–	–	–	No	1546.34	D5	337.30	D5
28	15	16	9	2	5	–	–	No	2477.09	D6	564.08	D5
29	15	4	2	2	–	–	–	No	3089.37	D7	**1.028.40**	D3
*30*	*10*	4	4	12	–	–	*9*	No	1288.11	D3	730.92	D3
32*	15	10	8	8	*22*	–	–	*Yes*	5037.96	D1	575.01	D5
*33*	*13*	5	5	2	–	–	–	No	**9122.67**	D3	684.32	D2
**EEG criteria missing, n = 7**												
1	15	16	16	16	0	0	0	–	4467.16	D4	245.92	D5
*8*	*14*	*15*	16	16	8	3	3	–	1338.07	D5	147.36	D5
*16*	*14*	*14*	16	*14*	*15*	8	*12*	–	3605.58	D2	145.02	D1
*18*^*†*^	*11*	3	4	4	–	–	–	–	**5173.93**	D1	776.71	D1
*21*	*12*	*6*	*6*	*7*	–	–	–	–	3683.31	D2	459.78	D6
*22*	*14*	2	1	3	–	–	–	–	301.52	D5	228.43	D3
*31*	*8*	0	2	2	–	–	–	–	**6283.33**	D7	366.87	D1

*SAE* septic-associated encephalopathy, *GCS* Glasgow Coma Scale, *FOUR* full outline of unresponsiveness scale, *D1–D2–D3* Days 1, 2, and 3 of evolution, *CAPD* Cornell Assessment of Pediatric Delirium, *EEG suggestive of SAE* electroencephalogram suggestive of septic-associated encephalopathy, *NSE* neuron specific enolase, *S100B* S100 calcium-binding protein B.

*Patient with clinical and EEG findings suggestive of SAE.

†The only deceased patient.

^a^Values in italics represent abnormal results presumptive of sepsis-associated encephalopathy.

^b^Values in italics represent a score disproportionate to the level of sedation used. Please, see Table S3 in Supplementary File 1 for more details.

^c^For EEG changes considered suggestive of SAE (“Yes”), please, see Table 3, and for all EEG results, please consult Table S5 in Supplementary File 1.

^d^Cells in bold represent levels above reference value.

Significant values are in italics.

Twenty-five patients (75.8%) met clinical criteria for SAE; 18 (54.5%) had reduced level of consciousness; 12 of 19 (63.2%) had delirium, and 7 of 26 (26.9%) had EEG changes suggestive of acute encephalopathy. NSE or S100B values were above the reference levels in 11 of 33 (33.3%) (Table 2). Twenty-six patients had the two diagnostic components (clinical criteria and EEG). In this group, 20 (76.9%) met at least one criterion, six (23.1%) met both criteria, and six (23.1%) met no one (considered non-SAE) (Table 2).

### EEG and biomarkers

**Figure 1:**
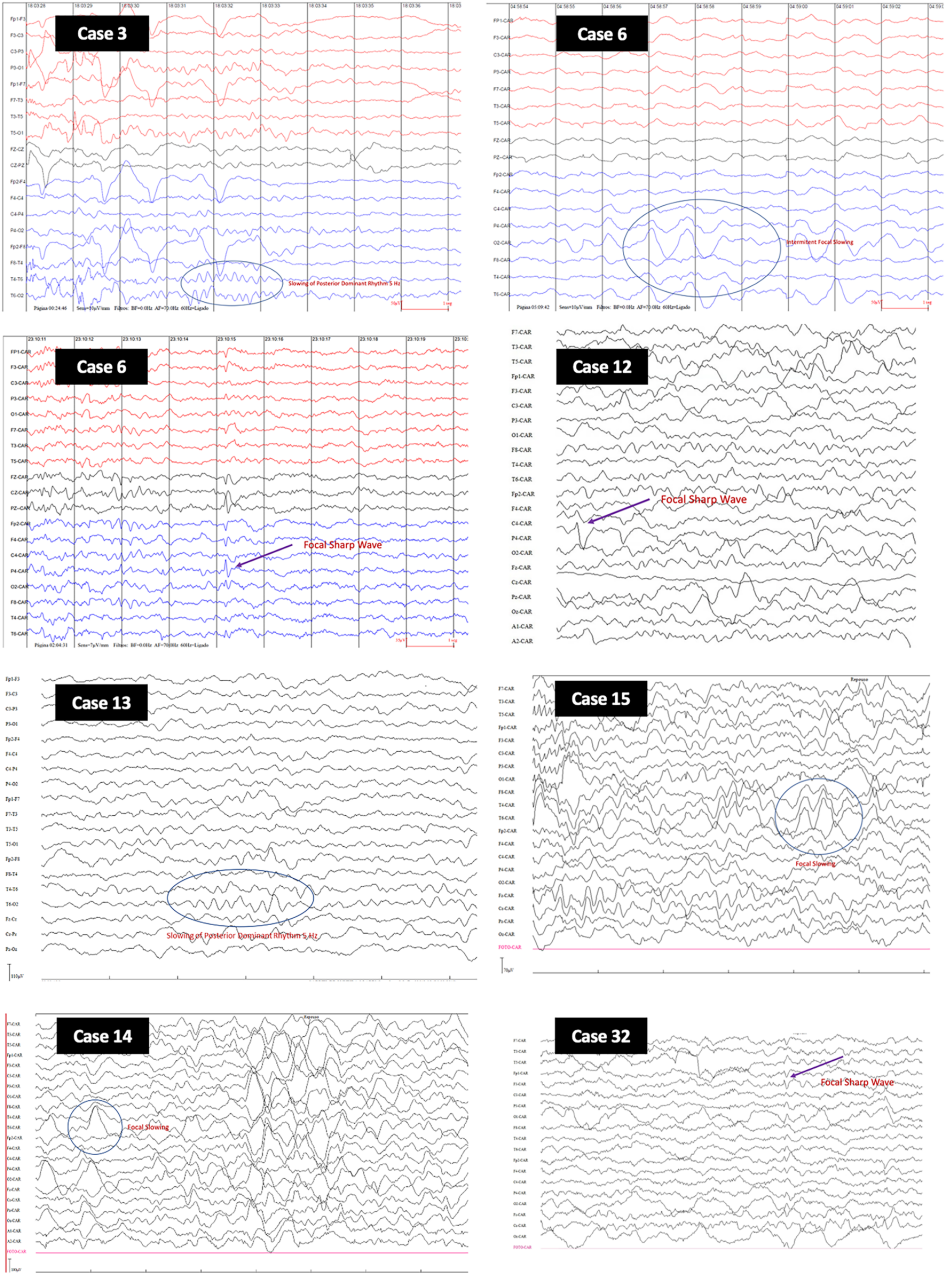
Some selected images from the electroencephalogram of seven patients highlighting findings considered suggestive of encephalopathy associated with sepsis.

Table 3 shows the seven patients with EEG suggestive of encephalopathy: four (57.1%) showed interictal epileptiform discharges, three (42.9%) showed intermittent focal slowing, and two (28.6%) showed both abnormalities. Figure 1 presents some representative EEG images of these patients. The EEG results for all patients are shown in Supplementary Table S6 online. Overall, we found a clear predominance of delta and theta waves in 96% of patients as the background rhythm.

**Table 3:** Electroencephalographic characteristics considered presumptive of sepsis-associated encephalopathy in seven patients.

Patient no.	Day	Sedative drugs and doses^a^	RASS	NMB	Anticonvulsant	EEG duration (minutes)	Physiological sleep elements^b^	Continuous slowing	Intermittent slowing	Predominant rhythm	Periodic epileptiform discharges	TW
3	D6	None	0	No	No	37	N/A, awake	Yes, diffuse	No	Theta	No	No
6	D7	1 (0.3)/2 (3.2) 3 (0.5)/4 (0.05)	−4	No	No	720	1, 2, 3	Yes, diffuse	Yes, parietal, occipital, right	Delta	Yes, frontal, parietal, occipital, right	No
12	D4	3 (0.1)	1	No	No	70	Absent	Yes, diffuse	Yes, central and parietal, right	Delta/Theta	Yes, central, parietal	No
13	D2	None	0	No	No	422	Spindles, bilateral	Yes, diffuse	No	Delta/Theta	No	No
14	D6	1 (0.06)/2 (0.6) 3 (0.09)	−1	No	No	600	Spindles, bilateral	Yes, diffuse	No	Delta/Theta	Yes, frontal	No
15	D3	1 (0.07) 2 (0.78)/3 (0.6)	−3	No	Phenobarbital, phenytoin	364	Spindles, bilateral	Yes, diffuse	Yes, temporal, right	Delta/Theta	No	No
32	D4	1 (0.3)/2 (3.1) 3 (0.14)/4 (0.27)	−5	Yes	No	117	Spindles, right	Yes, diffuse	No	Delta/Theta	Yes, frontal	No

*RASS* Richmond Agitation Sedation Scale, *NMB* neuromuscular block, *EEG* electroencephalogram, *TW* triphasic waves.

^a^1 Midazolam (mg/kg/h); 2 Fentanyl (µg/kg/h); 3 Dexmedetomidine (µg/kg/h); 4 Ketamine (mg/kg/h).

^b^1 spindles, 2 vertex sharp-waves, 3 K-complex.

**Figure 2:**
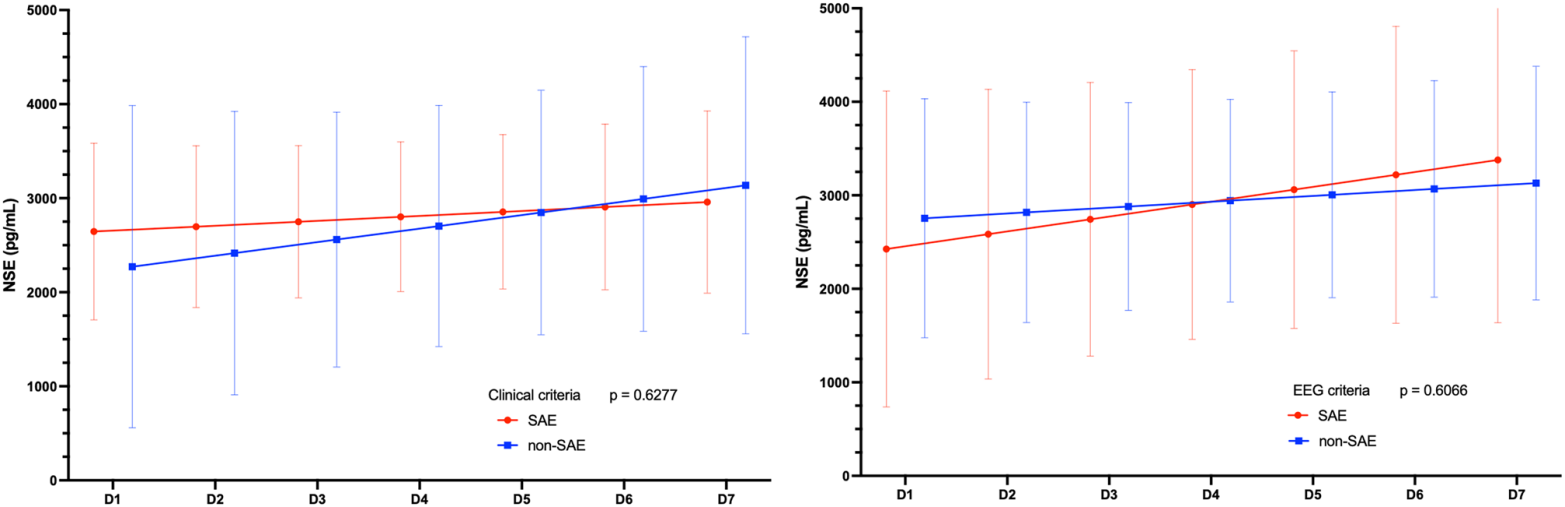
Temporal curves of predicted means of the Neuron-Specific Enolase (NSE) biomarker in the groups with and without clinical criteria (on the lower left) and with and without ECG criteria (on the lower right) for sepsis-associated encephalopathy in the first seven days after the diagnosis of sepsis.

**Figure 3:**
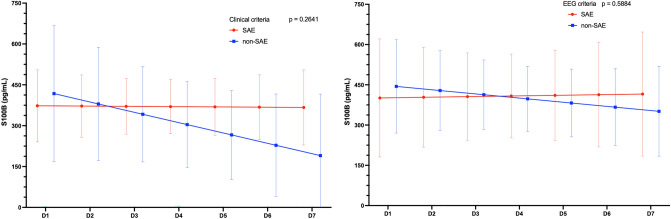
Temporal curves of predicted means of the S100 calcium-binding protein B (S100B) biomarker in the groups with and without clinical criteria (on the lower left) and with and without ECG criteria (on the lower right) for sepsis-associated encephalopathy in the first seven days after the diagnosis of sepsis.

No differences in time course of plasma NSE levels were observed between groups with and without clinical (p = 0.63) or EEG (p = 0.61) criteria, adjusting for age and Pediatric Index of Mortality 3 (PIM3) score (Fig. 2 and Supplementary Table S2 online). Changes in plasma S100B levels over time also did not differ between groups with and without clinical (p = 0.26) or EEG (0.59) criteria, adjusting for age and PIM3 at admission (Fig. 3 and Supplementary Table S3 online).

Table S5 shows the evolution of C-reactive protein on days 1, 3, and 7 in 25 patients in whom this result was available. Eleven patients (44%) had a serum level ≤ 8 mg/dL on D1. Among these, five (45.45%) did not develop clinical or electroencephalographic signs of SAE, four (36,4%) had only clinical features of SAE, and two (18,2%) had both clinical and EEG signs of SAE. Among the 17 with values above this cut-off point, 16 (94.1%) developed SAE.

### Other clinical features and outcomes

Details on sedative drugs and levels of sedation, other clinical features (Pediatric Logistic Organ Dysfunction 2—PELOD-2, hemodynamic support, neurological examination, and isolated infectious agent), the PICU length of stay, and the results of FSS over time are presented in Supplementary Tables S4 and S5 online.

Among the six patients who fulfilled clinical and EEG SAE criteria, four (66,7%) had mild disease (patients #3, 12, 13, and 15) but only one organ failure at admission, no other neurological signs besides reduced level of consciousness or miosis and absence of eye contact, and a normal FSS during the four evaluation periods (at admission, at discharge, on 3- and 6-month follow-up). The other two (patients #14 and 32) had multiple organ failure and abnormal neurological signs. On the other hand, all six patients who did not meet any of the presumptive SAE-criteria had moderate to severe disease, with multiple organ failure and neurological examination abnormalities (Tables 2 and Supplementary Table S5 online).

Three patients (9.1%) had altered FSS at PICU discharge, and one of them (4%) still presented altered FSS three months after hospital discharge, but all had normal FSS at six months of follow-up (Supplementary Table S5 online). All three patients had clinical signs of SAE, but EEG considered normal (one had no EEG). Two of these patients had severe disease, with multiple organ failure. The only patient who died had clinical signs of SAE and an elevated level of NSE, but the EEG was unavailable (Table 2).

## Discussion

In this study, pediatric patients with sepsis were assessed for SAE based on clinical and electroencephalographic criteria. Biomarkers were analyzed longitudinally in all patients. The diagnosis varied from 23.1 to 75.8%, depending on the criteria used. Biomarkers did not prove to be helpful to differentiate SAE and non-SAE groups. Despite the severity of the patients and the diagnosis or not of SAE, the prognosis assessed six months after discharge using the functional status scale was favorable. Based on our findings, a pathognomonic pattern was not detected. Decreased level of consciousness and/or delirium should be the basis of the diagnosis. However, the level of sedation and possibly other factors may interfere with these clinical parameters and encephalographic criteria, making the diagnosis of SAE a major challenge. New consensual diagnostic definitions and mainly prognostic studies are needed.

There are only a few case reports on SAE in pediatrics^21–26^ and four studies with a greater number of patients^11,12,19,27^. In these studies, the criteria used for the diagnosis of SAE were fundamentally the reduction in the level of consciousness, assessed by the GCS^11,19,27^ or by the Full Outline of Unresponsiveness (FOUR) scale^27^ associated with alterations in the EEG^11,12,19^ or neuroimaging^11^, with a normal cerebrospinal fluid^19^. However, these studies did not consider the influence of sedative drugs, analgesics, or neuromuscular blockers on the level of consciousness, nor the diagnosis of delirium as suggestive of SAE.

In our study, we assessed the level of consciousness through the GCS only on admission, in non-intubated patients, and before the start of sedative drugs. On the following three days, we used the FOUR scale, trying to establish a proportionality between the degree of sedation and the change in the scale. In this line, patients using ketamine (significant dissociative effect^28^) and/or propofol (great hypnotic effect^29^) with scores < 16 were considered to have “proportional” changes in the scale due to their use. On the other hand, patients with a severely reduced level of consciousness, who were not using ketamine, propofol, or high doses of other sedatives, were considered to have a “disproportionate” reduction in the level of consciousness, probably due to SAE. We also considered delirium as part of the clinical diagnosis of SAE, as adopted by most adult studies^2–4,6^. We assess delirium in the first three days of sepsis, using the CAPD scale, which was validated in 2013, covers the entire pediatric age group of the study, and allows easy application at the bedside, with a sensitivity of 94.1% and specificity of 79.2%. However, about 1/3 of our patients were too sedated to have this scale applied.

Using only these clinical criteria, available in any intensive care service, our frequency of SAE was between 54.5% (only reduced level of consciousness) to 75.8% (also considering the presence of delirium). If we consider only delirium, our frequency (63.2%) is similar to that reported in studies with adult patients^4,6^. To date, no pediatric study has considered delirium, which is widely accepted as part of the diagnosis of SAE^2–6,14^. Hamed et al., using a GCS ≤ 12 as the SAE diagnostic criterion, reported that among 40 consecutive pediatric patients admitted with severe sepsis, 16 (40%) had SAE^11^. Considering that our clinical criteria for SAE included any value < 15 on the GCS at admission or values < 16 on the FOUR scale, when disproportionate to the degree of sedation used, our findings of 54.5% are next to that described by Hamed et al.^11^, the only pediatric study which permitted to estimate this frequency of decreased level of consciousness.

Only about a quarter of our patients had EEG alterations that could not be explained by the use of sedative medications and were consistent with acute encephalopathy. We cannot rule out the possibility that the other three-quarters of patients with EEG changes attributed to sedation may also have had SAE and our results are underestimated. However, we opted for a more conservative EEG analysis, and thus the percentage of SAE in our study by this criterion was lower than that described in the literature. Of the three pediatric studies that used the EEG, (1) Hamed et al.^11^ reported changes in all children with SAE but did not define these abnormalities; (2) Kaur et al.^19^ considered as typical of SAE only the absence of a non-convulsive status epilepticus, and (3) Hsu et al.^12^ described alterations in all patients diagnosed as SAE but recognized that they could be related to the use of sedatives, among other factors. Studies in adult patients also reported a high frequency (up to 80%) of electroencephalographic changes^4,6,30,31^. Differences in EEG morphology caused by age were considered in the analysis of the exams, although our sample was relatively uniform, with 85% of patients under three years of age. In our analysis, if we considered the predominant presence of theta (4–8 Hz) and/or delta (< 4 Hz) waves as characteristics of SAE, according to the recommendation of Young et al.^32^ and Synek^33^, we would also have EEGs suggestive of SAE in all, but one patient. However, we decided to restrict the interpretation of the EEG, considering as the effect of sedation the presence of continuous or intermittent diffuse slowing, absence of EEG graphoelements of sleep, and predominant theta and delta waves as the background rhythm, if there were no other abnormal findings, such as intermittent or continuous focal slowing, interictal epileptiform discharges, periodic patterns, electrographic seizures or triphasic waves. Therefore, our findings of abnormalities compatible with SAE, such as intermittent focal slowing (43%) and/or interictal epileptiform discharges (57%), became proportionally higher, but when we considered all patients with an EEG, they were similar (27% and 15%, respectively) to those reported in a systematic review by Hosokawa et al.^34^. Interestingly, one of the seven patients with an altered EEG (patient #6) by our restricted criteria did not have a clinical diagnosis of SAE. A possible explanation is that the reduction in the level of consciousness assessed by the FOUR scale was not “proportional” to the sedative medication used, showing that this attempt of isolating the effect of sedation from the assessment scales is not perfect.

Concerning the changes in biomarkers, one-third of our patients had abnormal levels of NSE or S100B at any time point in the first seven days of sepsis. NSE is the intracytoplasmic glycolytic enzyme enolase produced by neurons, and its increase is related to neuronal injury and neuron death. S100B is a calcium-binding protein produced in the central nervous system by astroglial cells. Although both have been used as markers of brain injury in adults with SAE^9,10^, their abnormal levels may be related to critical illness per se^3^, with low sensibility and specificity for the diagnosis of SAE, limiting their clinical relevance^6^ and their use in the diagnosis criteria. However, three of the four pediatric studies evaluating SAE used these biomarkers. For these reasons, we decided to include them in our study, but not as part of the diagnosis criteria, given the limitations already mentioned. One of these studies, by Hsu et al.^12^, found that NSE and S100B were elevated on each of the first seven days after diagnosis of septic shock in children compared to controls (febrile children without sepsis), which was interpreted as neurological injury markers. Nevertheless, the authors reported the average plasma levels of biomarkers per day but did not assess differences between groups with and without clinical or EEG criteria for SAE. We used this study to define our cutoff values, however, we were unable to demonstrate the same proportion of abnormal values. None of our patients had both markers altered. Three of the eleven patients with values above the cutoff point did not even have clinical or EEG changes, but the small number of patients limits interpretation of these data. Our study also failed to demonstrate significant differences in the temporal evolution of these biomarkers in the first seven days of sepsis between the groups considered with and without SAE. These findings were independent of age, mortality risk at admission (PIM3), and even the biomarkers reference levels used. The groups presented statistically similar evolutionary levels regardless of the cut-off points used as reference. The time of sepsis in which the biomarkers were collected can also influence serum levels. In our study, patients were included in the initial phase, 75% with less than four hours of evolution. This was probably influenced by the fact that the three hospitals in the study belonged to the same hospital network, and the team used the same sepsis protocol, directing all these patients to the PICU as soon as possible.

As for the nonspecific biomarker, C-reactive protein, our data showed that high values (≥ 8 mg/dL) on the first day of sepsis were associated with a high probability of developing SAE. This reinforces the link between the intensity of the inflammatory state at the onset of sepsis and the possibility of developing encephalopathy.

The clinical course (severity, length of stay, and mortality) of our patients with and without SAE did not differ. The only patient with SAE who died had a decreased level of consciousness on admission, reduced FOUR scale (considered proportional to the level of sedation), could not undergo delirium assessment due to the level of sedation, did not undergo EEG, but presented an increased level of NSE. The mortality rate of pediatric patients with SAE differs from adult patients^2,4,6,14^. Other pediatric case reports and cohort studies showed good results and prognoses^22,23,25,26^ like ours, even though some studies have high mortality^12,24,27^. The controversies between these studies may be attributed to the sample size, clinical and EEG criteria. The functional alterations identified by the FSS in three patients at discharge disappeared at six months of follow-up, in contrast to the findings by Kaur et al., who demonstrated potential long-term neurodevelopmental impairment in up to half of the patients studied^19^.

This study has some strengths but several limitations. As strengths, we highlight the judicious criteria to characterize the clinical and EEG features of SAE. This is the only study that made efforts to rule out the effects of sedation on the clinical and EEG criteria used to diagnose SAE. The EEG criteria we used for the SAE diagnosis (especially the focal findings) was very restricted and might have resulted in underestimation. However, we considered this unprecedented option more of a strength than a limitation, as we intended to avoid the confounding factor caused by the level of sedation. As to the strategy used to differentiate decreased level of consciousness as proportional or not to the level of sedation used, we recognize that this method may not be readily generalizable, perhaps being more applicable in a research setting than in clinical practice. Despite that, it is worth noting that in our study, about half of the patients already had an altered GCS on admission before starting sedative medications and already met the clinical criteria for SAE, independent of sedation.

As limitations, we highlight the following points: (a) the small sample size, representativeness, and generalizability: We had a low rate of enrollment (33%). The resulting small sample size allowed only an exploratory data analysis with reduced precision. Nevertheless, these findings contribute to the scarce literature on SAE in pediatric patients. Our sample is made up of patients from three hospitals in Rio de Janeiro and may not be representative of the Brazilian population. However, on the other hand, the findings we described are similar to those already reported in different parts of the world, such as the USA^12^, India^19^, and Egypt^11,27^, of studies with samples of similar size to ours, suggesting that our findings may be generalizable; (b) the time frame of the study and the lack of concordance of EEG and biomarkers assessment. Initially, we chose to limit the study to the first three days of sepsis evolution, as this period coincides with the peak of the pro-inflammatory cascade and, probably, with the highest intensity of pathophysiological phenomena associated with organ dysfunctions, including SAE. However, as we were unable to collect biomarkers regularly on these three days and we were also unable to obtain authorization to perform EEG in this period in most patients, we decided to extend the study until the seventh day for these two assessments; (c) approximately 20% of the patients did not have an EEG but we took this into account, excluding these patients when estimating the general frequency of SAE; (d) interruption of sedation to assess mental status: this would be ideal. However, most of our patients were young children (85% < 3 years) in the acute, more severe phase of the disease. Under these conditions, the clinical team did not agree with the brief interruption proposal. As the study was observational, the research team could not interfere with this approach. In fact, pediatric studies in this age group also report great difficulty (if not a contraindication) in stopping sedation in these patients. On the other hand, it should be noted that almost half of our patients already had a change in the level of consciousness at the time of inclusion in the study (GCS < 15) when they were not using sedatives; (e) biomarkers: although we planned to measure them at least on days 1, 2, 3, 5, and 7 in all patients, the number of samples and the day of collection in the first week varied due to logistical and structural issues. On the other hand, we tried to compensate for this through statistical analysis, as we conducted a temporal analysis which accounts for missing values, although at the expense of reduced precision. We also had great difficulty in finding in the literature what levels would be expected in non-septic children. We adopted as reference only one pediatric study similar to ours, which used a control group^12^, but these children were in a febrile state, which is far from ideal. All these issues may have resulted in the underestimation of altered biomarkers. Still concerning biomarkers, we could have studied other cytokines, procalcitonin, etc. Unfortunately, budgetary constraints made this impossible; (f) not all patients underwent cerebrospinal fluid examination to rule out meningitis or encephalitis definitively, since this exam was indicated only in patients with high clinical suspicion. Moreover, the ethical committee did not allow the realization of this exam as a routine. Cerebrospinal fluid analysis was altered in 21 patients who were excluded from the study; (g) follow-up and long-term outcomes: unfortunately, it was impossible to conduct presential follow-up visits with a more complete neurological and neurobehavioral assessment for logistical reasons and budgetary limitations. Although it was not ideal, we performed the FSS scale at three and six months after discharge, demonstrating short-term changes in almost 10% of patients but fortunately reversible; (h) finally, due to difficulties in mobilizing patients to the radiology sector and possible parents' refusal to consent to the necessary anesthesia, MRI was not performed.

In the current study, a definitive diagnostic pattern for SAE remained unclear. Lower levels of consciousness and/or delirium should continue to be the basis for SAE diagnosis, but sedation and possibly other factors may be a significant confounder, also to EEG interpretation. The role of NSE and S100B requires a better definition and cannot be used in the diagnosis at present. Therefore, the diagnosis of SAE in pediatric patients remains a major challenge. It may be impossible to individualize SAE from other sepsis organ dysfunctions. Although new consensual diagnostic definitions for SAE in pediatrics are needed, it is perhaps more important that future research focuses more on long-term neurologic and neurobehavioral impairment in all patients with neurologic symptoms associated with sepsis.

## Methods

### Design, settings, and study population

This is an observational, prospective, longitudinal study conducted in three PICUs in Rio de Janeiro, Brazil. Data were collected from April 2017 to March 2019. Patients from 1 month to 18 years of age, consecutively admitted with a diagnosis of severe sepsis, according to the international consensus conference on pediatric sepsis^35^, were considered eligible. The exclusion criteria were: family refusal to sign the informed consent; the impossibility of obtaining consent in the first 72 h of sepsis diagnosis; admission to palliative care; history of exposure to drugs, toxic substances, alcohol, industrial agents, heavy metals, or any substance known to cause a change in the level of consciousness; acute diseases of the central nervous system with repercussions on cognitive function, such as meningoencephalitis by any agent (viruses, fungi or bacteria) or demyelinating syndromes; epilepsy; non-progressive chronic encephalopathy; primary immunodeficiency; cancer undergoing chemotherapy; regular use (> 2 weeks) of immunosuppressants in the previous six months; use of steroids in immunosuppressive doses in the previous three months, and use of neuroleptic drugs with cognitive repercussions, for instance in the case of psychiatric diseases. Other exclusion criteria after entry into the study were patients who presented any change in the level of consciousness caused by clinical events such as intracranial hemorrhage, hypoxic-ischemic encephalopathy after an episode of cardiac arrest, acute hypoglycemia (< 60 mg/dL), severe hyponatremia (Na < 125 mEq/L) or hypernatremia (Na > 155 mEq/L).

### Data collection

Upon admission to the PICU, all patients had the following data collected: (a) demographics (b) comorbidities; (c) infection site; (d) Pediatric Index of Mortality 3 (PIM3)^36^; (e) Pediatric Logistic Organ Dysfunction 2 (PELOD-2)^37^; (f) Glasgow Coma Scale (GCS); and (g) Functional Status Scale (FSS)^38^. The following data were assessed daily: (a) neurological examination; (b) Full Outline of Unresponsiveness (FOUR) scale^39^; (c) Cornell Assessment of Pediatric Delirium (CAPD) scale^40^; (d) Richmond Agitation-Sedation Scale (RASS) and State Behavioral Scale (SBS)^41,42^; (e) use and doses of sedative drugs; (f) use of neuromuscular blockers and anticonvulsants medications; (g) PELOD-2 score; and (h) need for hemodynamic support drugs. An EEG lasting 30 min to 12 h was performed between days 1 and 7 of the diagnosis of sepsis. At least one blood sample was collected to measure the neurological biomarkers NSE and S100B within the first week of diagnosis. The causal agent of sepsis, PICU length of stay, and mortality were also recorded. Upon discharge from the PICU, the FSS was applied again, and a follow-up call was made three and six months after hospital discharge when the FSS was repeated.

### Presumptive diagnosis of sepsis-associated encephalopathy

The diagnosis of SAE was considered as probable whenever one of the following conditions was present: (a) decreased level of consciousness on admission (GCS < 15); (b) decreased level of consciousness (FOUR scale < 16) between days 1 (D1) and 3 (D3) in patients who were not using sedative, analgesic or anticonvulsant drugs, or when this decrease in the level of consciousness was considered disproportionate to the level of sedation used, as in the following situations: (i) mild level of sedation (RASS = −1) when using low doses of sedatives, except ketamine or propofol; (ii) deep level of sedation (RASS ≤ −3 or SBS ≤ −2) in patients who were not using ketamine, propofol, or high levels of any other sedative drug or neuromuscular block; (c) presence of hyperactive or hypoactive delirium (CAPD ≥ 9) between D1 and D3; (d) EEG suggestive of acute encephalopathy between D1 and D7.

Patients with a moderate level of sedation (RASS = −2 or SBS = –1) and/or using ketamine or propofol with a score < 16 on the FOUR scale were considered to have a decreased level of consciousness proportional to the level of sedation and, therefore, were not considered as a probable diagnosis of SAE by this criterion.

### Electroencephalogram

The EEG was registered in a 32-channel digital electroencephalograph from Neurotec (Neurotec, Itajubá, MG, Brazil), using the Neuromap 40i version 2013 software, or from EMSA (EMSA, Rio de Janeiro, RJ, Brazil), using the TELAS software. We used the complete 10–20 electrode placement system, time constant of 0.3 s, sampling of 256 pcs, impedance < 5 kOhm and low pass filter of 70 Hz and high pass of 0.5 Hz, sensitivity of 10 uV/mm, and speed of 3 cm/s.

The following variables were analyzed: (a) presence of a posterior dominant rhythm of wakefulness; (b) presence of graphoelements of sleep; (c) predominant frequencies in the background rhythm; (d) continuous or intermittent focal slowing and its location; (e) presence of interictal epileptiform discharges; (f) presence of electrographic seizures; (g) presence of periodic patterns. It was not possible to state the presence of abnormalities in the following situations due to the confounding factor of medications: (i) slowing or absence of the posterior rhythm of wakefulness; (ii) absence of graphoelements of sleep; and (iii) continuous or intermittent diffuse slowing of the background rhythm.

EEG was considered abnormal and suggestive of acute encephalopathy when one or more of the following findings were present: (a) asymmetry of the posterior dominant rhythm; (b) fixed asymmetry of graphoelements of sleep; (c) presence of continuous or intermittent focal slowing; (d) presence of interictal epileptiform discharges; (e) presence of electrographic seizures; and (f) presence of periodic patterns.

### Biomarkers

To evaluate the biomarkers, 1.0 mL of blood was collected in sodium citrate tubes. After centrifugation (within the first two hours), the plasma was stored at −80 ºC until use. NSE and S100B were measured using specific enzyme-linked immunosorbent assay kits (DuoSet ELISA Development System DY5169-05, R&D Systems, Minneapolis, MN, USA), according to the manufacturer’s instructions. The considered reference levels were those reported by Hsu et al. for children with an acute febrile disease but without sepsis and were 2700–5100 pg/mL for NSE and 700–900 pg/mL for S100B^12^.

### Data processing and statistical analysis

Clinical data and biomarker concentration results were recorded on electronic case report forms. EEGs were digitally recorded for further analysis by one of the researchers (RSF), a neuro pediatrician, specialist in brain electrophysiology, blinded to clinical suspicion of SAE by the clinical and research team.

Sample size was determined by the study period (convenience). Continuous variables are presented as means and standard deviations or medians and interquartile ranges, while categorical variables as proportions. Differences in time course of biomarkers plasma levels between groups with and without criteria for SAE (clinical or electroencephalographic) were analyzed using linear mixed-effect models, which assesses the rate of change over time by the group-by-time interaction variable, accounting for correlations between repeated measures over time and incomplete data tracking. Parametric curves were fitted to the data. For both diagnostic criteria (clinical or electroencephalographic) and both biomarkers (NSE and S100B) the most parsimonious models accounted for a linear change over time, including the following variables: time, group, and time*group. The four models were adjusted for age and probability of death on admission (PIM3). All models were fitted using random intercept, and we assumed an unstructured variance–covariance pattern (covariance matrix G). Residual analyses of all models were performed, and their distributions showed the adequacy of the fitted models. Longitudinal analyses were performed using SAS OnDemand for Academics (SAS Inc., Cary, NC, USA), and the other statistical analysis using the SPSS software, version 24.0 (IBM Corp. Armonk, NY, USA). Statistical significance was set at p < 0.05.

### Ethics approval, consent to participate, and methods guidelines

The study protocol was approved by the Research Ethics Committee of D’Or Institute for Research and Education (CAAE, No. 50603015.5.0000.5249). All families of patients included gave their signed informed consent to participate. All methods were performed in accordance with the Declaration of Helsinki. The study followed the STROBE Statement guidelines for reporting observational cohort studies (https://www.strobe-statetment.org).

## Supplementary Information

Supplementary Information.
