# Supplementary material for: Clinical features, electroencephalogram, and biomarkers in pediatric sepsis-associated encephalopathy
Source: Sci Rep. 2022 Jun 23;12:10673. doi: 10.1038/s41598-022-14853-z (PMC9225983; doi:10.1038/s41598-022-14853-z)
Supplement: Supplementary file 1 — Supplementary Information. [file 41598_2022_14853_MOESM1_ESM.docx]

**Clinical features, electroencephalogram, and biomarkers in pediatric sepsis-associated encephalopathy**

Bruno Espírito Santo de Araújo, Rosiane da Silva Fontana, Maria Clara de Magalhães-Barbosa, Fernanda Lima-Setta, Vitor Barreto Paravidino, Margarida dos Santos Salú, Mariana Barros Genuíno-Oliveira, Jaqueline Rodrigues Robaina, Antonio José Ledo Alves da Cunha, Fernanda Ferreira Cruz, Patrícia Rieken Macedo Rocco, Fernando Augusto Bozza, Hugo Caire de Castro-Faria-Neto, Arnaldo Prata-Barbosa.

**Corresponding author**

Arnaldo Prata-Barbosa

D’Or Institute for Research & Education (IDOR), Department of Pediatrics

Rua Diniz Cordeiro 30 – Botafogo, Rio de Janeiro, RJ, 22281-100, Brazil.

arnaldo.prata@idor.org

**Figure S1.** Number of eligible patients, reasons for exclusion, and patients included in the study, by center, and in total. Each center contributed to the study in different periods from April 2017 to March 2019.


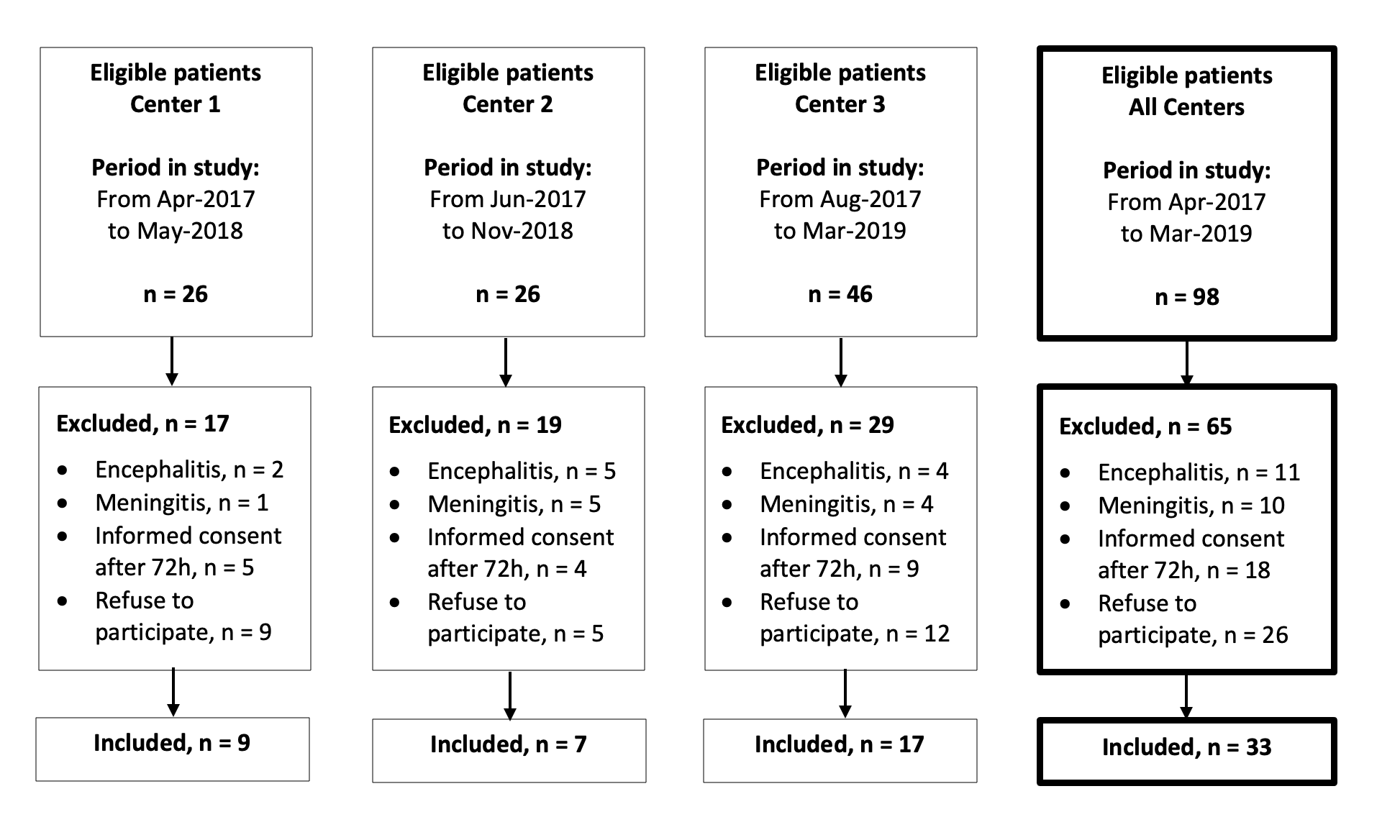


**Table S1.** Patient demographics, time of sepsis at study inclusion, length of PICU stay, and outcome (continue next page).

| **No.** | **Birth date** | **Age** | **Sex** | **Color** | **Weight (Kg)** | **Hospital admission date** | **Sepsis diagnosis (date)** | **Sepsis diagnosis (hour)** | **PICU admission date** | **PICU admission (hour)** | **Time with sepsis at PICU entry** | **PICU discharge date** | **PICU LOS (days)** | **PICU outcome** | **Discharge date** | **Hospital outcome** |
| --- | --- | --- | --- | --- | --- | --- | --- | --- | --- | --- | --- | --- | --- | --- | --- | --- |
| 1 | 28-Dec-01 | 15y, 3mo | F | White | 51,0 | 05-Apr-17 | 05-Apr-17 | 17:00 | 05-Apr-17 | 17:02 | 0:02 | 11-Apr-17 | 7 | Discharge | 19-Apr-17 | Discharge |
| 2 | 19-Sep-14 | 2y, 8mo | F | Black | 13,0 | 05-Jun-17 | 05-Jun-17 | 23:30 | 06-Jun-17 | 0:40 | 1:10 | 18-Jun-17 | 13 | Discharge | 25-Jun-17 | Discharge |
| 3 | 20-Apr-16 | 1y, 1mo | F | White | 10,0 | 07-Jun-17 | 07-Jun-17 | 22:20 | 07-Jun-17 | 23:50 | 1:30 | 16-Jun-17 | 10 | Discharge | 16-Jun-17 | Discharge |
| 4 | 14-Jun-16 | 1y, 1mo | M | Black | 10,3 | 21-Jul-17 | 21-Jul-17 | 21:00 | 21-Jul-17 | 22:00 | 1:00 | 08-Aug-17 | 19 | Discharge | 14-Aug-17 | Discharge |
| 5 | 15-Jul-15 | 2y, 0mo | F | White | 9,7 | 12-Aug-17 | 12-Aug-17 | 14:00 | 12-Aug-17 | 18:10 | 4:10 | 30-Aug-17 | 19 | Discharge | 03-Sep-17 | Discharge |
| 6 | 19-May-15 | 2y, 4mo | F | White | 13,3 | 23-Sep-17 | 23-Sep-17 | 12:30 | 23-Sep-17 | 16:00 | 3:30 | 09-Oct-17 | 17 | Discharge | 18-Oct-17 | Discharge |
| 7 | 7-Oct-14 | 3y, 1mo | F | White | 12,0 | 08-Nov-17 | 08-Nov-17 | 6:00 | 08-Nov-17 | 6:02 | 0:02 | 30-Nov-17 | 23 | Discharge | 08-Dec-17 | Discharge |
| 8 | 7-Dec-17 | 3mo | M | White | 4,8 | 01-Apr-18 | 03-Apr-18 | missing | 03-Apr-18 | 22:00 | missing | 06-Apr-18 | 4 | Discharge | 14-Apr-18 | Discharge |
| 9 | 20-Jun-16 | 1y, 10mo | M | Black | 12,9 | 14-May-18 | 15-May-18 | 21:40 | 16-May-18 | 5:30 | 7:50 | 08-Jun-18 | 24 | Discharge | 12-Jun-18 | Discharge |
| 10 | 11-Jun-16 | 1y, 0mo | F | Black | 9,0 | 28-Jun-17 | 28-Jun-17 | 19:15 | 28-Jun-17 | 19:30 | 0:15 | 18-Jul-17 | 21 | Discharge | 26-Jul-17 | Discharge |
| 11 | 20-Apr-16 | 1y, 6mo | M | White | 11,0 | 20-Oct-17 | 20-Oct-17 | 10:00 | 20-Oct-17 | 11:30 | 1:30 | 04-Nov-17 | 16 | Discharge | 11-Nov-17 | Discharge |
| 12 | 31-Mar-16 | 1y, 9mo | M | White | 11,5 | 28-Jan-18 | 28-Jan-18 | 17:00 | 28-Jan-18 | 20:40 | 3:40 | 19-Feb-18 | 23 | Discharge | 26-Feb-18 | Discharge |
| 13 | 11-Jul-15 | 2y, 8mo | M | White | 12,0 | 21-Mar-18 | 22-Mar-18 | 19:00 | 22-Mar-18 | 19:00 | 0:00 | 28-Mar-18 | 7 | Discharge | 29-Mar-18 | Discharge |
| 14 | 15-Mar-12 | 6y, 0mo | F | Black | 23,0 | 29-Mar-18 | 29-Mar-18 | 12:00 | 29-Mar-18 | 12:45 | 0:45 | 12-Apr-18 | 15 | Discharge | 29-Apr-18 | Discharge |
| 15 | 3-Jul-17 | 1y, 1mo | F | Black | 9,1 | 24-Aug-18 | 24-Aug-18 | 10:00 | 24-Aug-18 | 14:00 | 2:00 | 31-Aug-18 | 8 | Discharge | 02-Sep-18 | Discharge |
| 16 | 11-Jul-16 | 2y, 4mo | F | White | 14,0 | 11-Nov-18 | 11-Nov-18 | 14:50 | 12-Nov-18 | 14:50 | 0:00 | 20-Nov-18 | 9 | Discharge | 06-Dec-18 | Discharge |
| 17 | 14-Jul-16 | 1y, 1mo | F | Black | 9,5 | 15-Aug-17 | 30-Aug-17 | 12:00 | 30-Aug-17 | 20:12 | 8:12 | 17-Sep-17 | 19 | Discharge | 23-Sep-17 | Discharge |
| 18 | 9-May-17 | 3mo | F | White | 6,5 | 04-Sep-17 | 04-Sep-17 | 23:40 | 04-Sep-17 | 23:40 | 0:00 | 20-Sep-17 | 17 | Death | 20-Sep-17 | Death |
| 19 | 18-May-15 | 2y, 4mo | F | Black | 11,0 | 26-Sep-17 | 26-Sep-17 | 1:20 | 26-Sep-17 | 4:41 | 1:21 | 14-Nov-17 | 50 | Discharge | 29-Nov-17 | Discharge |
| 20 | 26-May-15 | 2y, 4mo | M | Black | 14,6 | 08-Oct-17 | 08-Oct-17 | 23:00 | 08-Oct-17 | 23:01 | 0:01 | 22-Oct-17 | 14 | Discharge | 24-Oct-17 | Discharge |
| 21 | 6-Aug-17 | 2mo | M | White | 5,6 | 20-Oct-17 | 20-Oct-17 | 22:30 | 21-Oct-17 | 1:05 | 2:35 | 31-Oct-17 | 11 | Discharge | 01-Nov-17 | Discharge |

PICU – Pediatric Intensive Care Unit, LOS – length of stay

**Table S1 (continued from the previous page).** Patient demographics, time of sepsis at study inclusion, length of PICU stay, and outcome.

| 22 | 10-Mar-17 | 8mo | M | Black | 8,4 | 18-Nov-17 | 19-Nov-17 | 18:00 | 20-Nov-17 | 0:01 | 6:01 | 18-Dec-17 | 29 | Discharge | 22-Dec-17 | Discharge |
| --- | --- | --- | --- | --- | --- | --- | --- | --- | --- | --- | --- | --- | --- | --- | --- | --- |
| 23 | 15-Jun-17 | 9mo | M | White | 8,5 | 08-Apr-18 | 08-Apr-18 | 14:00 | 08-Apr-18 | 17:38 | 3:38 | 23-Apr-18 | 16 | Discharge | 04-May-18 | Discharge |
| 24 | 18-Dec-17 | 7mo | F | White | 7,0 | 30-Jul-18 | 30-Jul-18 | 19:00 | 30-Jul-18 | 20:00 | 1:00 | 13-Aug-18 | 15 | Discharge | 20-Aug-18 | Discharge |
| 25 | 29-Aug-17 | 11mo | F | Black | 10,0 | 04-Aug-18 | 04-Aug-18 | 17:00 | 04-Aug-18 | 17:21 | 0:21 | 25-Aug-18 | 22 | Discharge | 28-Aug-18 | Discharge |
| 26 | 11-Dec-15 | 2y, 9mo | F | White | 14,0 | 22-Sep-18 | 22-Sep-18 | 21:30 | 23-Sep-18 | 4:00 | 6:30 | 03-Oct-18 | 11 | Discharge | 08-Oct-18 | Discharge |
| 27 | 5-Sep-17 | 1y, 0mo | F | Black | 8,4 | 24-Sep-18 | 24-Sep-18 | 22:00 | 25-Sep-18 | 2:48 | 4:48 | 12-Oct-18 | 18 | Discharge | 21-Oct-18 | Discharge |
| 28 | 14-Jan-16 | 2y, 9mo | M | Black | 13,5 | 14-Oct-18 | 14-Oct-18 | 22:00 | 14-Oct-18 | 23:30 | 1:30 | 13-Nov-18 | 29 | Discharge | 21/11/2018 | Discharge |
| 29 | 15-Dec-16 | 1y, 10mo | M | Black | 9,0 | 04-Nov-18 | 04-Nov-18 | 17:40 | 04-Nov-18 | 18:58 | 4:18 | 19-Nov-18 | 16 | Discharge | 03-Dec-18 | Discharge |
| 30 | 14-May-09 | 9y, 6mo | F | Black | 26,0 | 22-Nov-18 | 22-Nov-18 | 4:00 | 22-Nov-18 | 4:30 | 0:30 | 29-Nov-18 | 8 | Discharge | 30-Nov-18 | Discharge |
| 31 | 15-Jul-09 | 9y, 4mo | F | Black | 30,0 | 25-Nov-18 | 26-Nov-18 | 7:30 | 26-Nov-18 | 7:45 | 0:15 | 04-Jan-19 | 40 | Discharge | 12/01/2019 | Discharge |
| 32 | 2-Mar-18 | 11mo | F | White | 8,5 | 10-Feb-19 | 11-Feb-19 | 1:00 | 11-Feb-19 | 1:06 | 0:06 | 10-Mar-19 | 28 | Discharge | 18-Mar-19 | Discharge |
| 33 | 19-Sep-16 | 2y, 6mo | F | White | 19,4 | 26-Mar-19 | 26-Mar-19 | 18:20 | 26-Mar-19 | 22:00 | 3:40 | 20-Apr-19 | 26 | Discharge | 29-Apr-19 | Discharge |

PICU – Pediatric Intensive Care Unit, LOS – length of stay

**Table S2**. Neuron-Specific Enolase (NSE) mean values per day and estimated changes from baseline in the groups with and without clinical criteria and the groups with and without electrocardiographic criteria for sepsis-associated encephalopathy (SAE), comparing the temporal curves adjusted for age and death probability (assessed by PIM3).

| **Days** | **No. of patients** | **Raw NSE means per day** | **Estimated changes^a^ from baseline** | **No. of patients** | **Raw NSE means per day** | **Estimated changes from baseline** | **p-value**  **Time*group^b^** |
| --- | --- | --- | --- | --- | --- | --- | --- |
|  | **Without clinical criteria for SAE, n=8** | | | **With clinical criteria for SAE, n=25** | | |  |
| 1 | 1 | 930.86 | - | 10 | 3077.49 | - | 0.6277 |
| 2 | 4 | 3919.21 | 144,16 | 14 | 2618.13 | 52,23 |  |
| 3 | 6 | 2597.19 | 288,32 | 17 | 2557.51 | 104,46 |  |
| 4 | 4 | 2138.18 | 432,47 | 11 | 2818.39 | 156,69 |  |
| 5 | 7 | 2841.45 | 576,63 | 12 | 2362.88 | 208,93 |  |
| 6 | 4 | 3869.82 | 720,79 | 7 | 2987.74 | 261,16 |  |
| 7 | 3 | 2725.97 | 864,95 | 11 | 3137.76 | 313,39 |  |
|  | **Without ECG criteria for SAE, n=19** | | | **With ECG criteria for SAE, n=7** | | |  |
| 1 | 5 | 2902.87 | - | 3 | 2360.18 | - | 0.6066 |
| 2 | 8 | 3629.47 | 62,68 | 5 | 2153.84 | 158,76 |  |
| 3 | 13 | 2630.18 | 125,37 | 4 | 3567.04 | 317,51 |  |
| 4 | 9 | 2746.34 | 188,05 | 4 | 2683.11 | 476,27 |  |
| 5 | 11 | 2274.69 | 250,74 | 4 | 3748.47 | 635,03 |  |
| 6 | 8 | 3613.20 | 313,42 | 2 | 2539.41 | 793,79 |  |
| 7 | 7 | 2901.71 | 376,11 | 4 | 3346.61 | 952,55 |  |

PIM 3 - Pediatric Index of Mortality (version 3)

**^a^** Difference of the predicted means of each day in relation to the predicted mean of the first day.

**^b^**Adjusted by age and death probability (PIM3)

**Table S3.** S100 calcium-binding protein B (S100B) mean values per day and estimated changes from baseline, comparing the groups with and without clinical criteria and the groups with and without electrocardiographic criteria for sepsis-associated-encephalopathy (SAE), comparing the temporal curves adjusted for age and death probability (assessed by PIM3).

| **Days** | **No. of patients** | **Raw S100B means per day** | **Estimated changes^a^ from baseline** | **No. of patients** | **Raw S100B means per day** | **Estimated changes from baseline** | **p-value**  **Time*group^b^** |
| --- | --- | --- | --- | --- | --- | --- | --- |
|  | **Without clinical criteria for SAE, n=8** | | | **With clinical criteria for SAE, n=25** | | |  |
| 1 | 1 | 80.43 | - | 9 | 292.21 | - | 0.2641 |
| 2 | 4 | 316.69 | -37,93 | 14 | 307.22 | -1,00 |  |
| 3 | 6 | 363.43 | -75,86 | 17 | 456.38 | -2,01 |  |
| 4 | 4 | 396.81 | -113,79 | 11 | 428.08 | -3,01 |  |
| 5 | 7 | 332.02 | -151,72 | 12 | 333.88 | -4,02 |  |
| 6 | 4 | 174.13 | -189,64 | 7 | 311.56 | -5,02 |  |
| 7 | 3 | 42.76 | -227,57 | 11 | 340.49 | -6,03 |  |
|  | **Without ECG criteria for SAE, n=19** | | | **With ECG criteria for SAE, n=7** | | |  |
| 1 | 4 | 343.91 | - | 3 | 142.63 | - | 0.5884 |
| 2 | 8 | 528.74 | -15.48 | 5 | 415.75 | 2.42 |  |
| 3 | 13 | 433.72 | -30.96 | 4 | 484.93 | 4.84 |  |
| 4 | 9 | 332.55 | -46.44 | 4 | 575.05 | 7.26 |  |
| 5 | 11 | 261.16 | -61.92 | 4 | 523.53 | 9.68 |  |
| 6 | 8 | 303.17 | -77.40 | 2 | 164.23 | 12.10 |  |
| 7 | 7 | 343.91 | -92.88 | 4 | 233.25 | 14.52 |  |

PIM 3 - Pediatric Index of Mortality (version 3)

**^a^**Difference of the predicted means of each day in relation to the predicted mean of the first day

**^b^**Adjusted by age and death probability (PIM3)

Table S4 shows the sedation levels assessed by the RASS and SBS scales. The type of sedative and/or analgesic drugs and the mean doses received allowed us to define the relationship between the sedation level and the FOUR scale score, one of the criteria of presumptive SAE. The hatched cells in the FOUR scale column in both tables represent patients in whom the low scores (< 16) were considered disproportionate to the level of sedation observed, according to the type and doses of sedative and/or analgesic drugs they were receiving. In addition to the level of sedation and doses of sedative and analgesic drugs used, we also highlight the use of neuromuscular blockers and/or anticonvulsants, together with the EEG result, for a better correlation of these data.

In Table S5, we present other clinical features, especially signs of severity and organ dysfunction, represented by the PELOD-2 score and need for inotropic drugs, as well as the neurological examination of the first seven days of sepsis. This table also shows the isolated infectious agent, the PICU length of stay, and the results for the FSS scale applied on admission and discharge from the ICU and after three and six months of hospital discharge.

In Table S6, we present the EEG results of all patients. Overall, we found a clear predominance of delta and theta waves in 96% of patients as the background rhythm. In this table, we highlight the sedation level and doses of sedatives and analgesics used and the use of neuromuscular blockers and/or anticonvulsants on the EEG day to better correlate these data.

**Table S4.** FOUR scale scores, mean doses of sedatives and analgesics used, sedation level, use of neuromuscular blockers and anticonvulsants, and EEG results in patients with severe sepsis.

| **Patient^a^** | **FOUR scale^a^** | | | **Sedation and Analgesia^b,c^** | **RASS** | | | **SBS** | | | **Neuromuscular blocking** | | | **Anticonvulsant** | | | **EEG suggestive of SAE** |
| --- | --- | --- | --- | --- | --- | --- | --- | --- | --- | --- | --- | --- | --- | --- | --- | --- | --- |
|  | **D1** | **D2** | **D3** | mean doses D1-D3 | **D1** | **D2** | **D3** | **D1** | **D2** | **D3** | **D1** | **D2** | **D3** | **D1** | **D2** | **D3** |  |
| **All criteria available, n = 26** | | | | | | | | | | | | | | | | | |
| 2 | 10 | 13 | 12 | 1 (0.22) / 2 (1.39) / 3 (1.0) / **4 (2.4) / 5 (0.12)** | **-3** | **-1** | **-1** | **-2** | -1 | -1 | No | No | No | **Yes** | **Yes** | **Yes** | No |
| 3 | 16 | 16 | 16 | None | **1** | **0** | **0** | -1 | **0** | **0** | No | No | No | No | No | No | Yes |
| 4 | 16 | 8 | 5 | 1 (0.16) / 2 (2.07) / 3 (0.2) | **2** | **-4** | **-5** | **1** | -1 | **-3** | No | No | **Yes** | No | No | No | No |
| 5 | 0 | 2 | 2 | 1 (0.22) / 2 (1.94) / 3 (0.9) / **5 (0.21)** | **-5** | **-5** | **-5** | **-3** | **-2** | **-3** | **Yes** | **Yes** | No | No | No | No | No |
| 6 | 8 | 10 | 9 | 1 (0.31) / 2 (2.56) / **5 (0.17)** | **-4** | **-4** | **-3** | **-2** | **-2** | -1 | No | No | No | No | No | No | Yes |
| 7† | 9 | 10 | 2 | 1 (0.28) / 2 (1.84) / **4 (4.1)** / **5 (0.04)** | **-4** | **-4** | **-5** | **-2** | **-2** | **-2** | No | No | **Yes** | No | No | No | No |
| 9† | 1 | 1 | 10 | 1 (0.20) / 2 (1.95) / 3 (0.5) /**4 (4.8)** | **-5** | **-5** | **-3** | **-2** | **-2** | -1 | No | No | No | No | No | No | No |
| 10 | 5 | 7 | 9 | 1 (0.08) / 2 (0.62) / 3 (0.3) / **5 (0.26)** | **-3** | **-4** | **-3** | **-2** | **-2** | **-2** | No | No | No | No | No | No | No |
| 11 | 8 | 8 | 8 | 1 (0.08) / 2 (0.28) / 3 (0.1) / **5 (0.05)** | -4 | -4 | -4 | **-2** | **-2** | **-2** | No | No | No | No | No | No | No |
| 12 | 11 | 13 | 15 | 1 (0.03) / 2 (0.19) / 3 (0.11) / **5 (1.63)** | -2 | **0** | **1** | **-2** | **0** | **1** | No | No | No | No | No | No | Yes |
| 13† | 14 | 14 | 16 | None | **-1** | **0** | **0** | -1 | **0** | **0** | No | No | No | No | No | No | Yes |
| 14 | 8 | - | 10 | 1 (0.06) / 2 (0.75) / 3 (0.09) / **4 (1.7)** / **5 (0.02)** | **-3** | **-3** | -2 | **-2** | **-3** | -1 | No | No | No | No | No | No | Yes |
| 15 | 6 | 8 | 11 | 1 (0.06) / 2 (0.62) / 3 (0.2) / **4 (2.4)^d^** / **5 (0.60)^d^** | **-5** | **-4** | **-3** | **-2** | **-2** | -1 | No | No | No | **Yes** | **Yes** | **Yes** | Yes |
| 17† | - | 5 | 10 | 1 (0.30) / 2 (3.02) / **5 (0.84)** | **-4** | **-4** | **-3** | **-2** | **-2** | -1 | No | No | No | No | No | No | No |
| 19† | 9 | 8 | 6 | 1 (0.43) / 2 (4.28) / **5 (0.23)** | **-3** | **-3** | -2 | -1 | **-2** | -1 | No | No | No | No | No | No | No |
| 20 | 16 | 16 | 16 | 1 (0.03) | **0** | **-1** | **-1** | **0** | **0** | **0** | No | No | No | No | No | No | No |
| 23 | 5 | 5 | 5 | 1 (0.24) / 2 (2.01) / **5 (0.33)** | **-5** | **-5** | **-5** | **-3** | **-3** | **-3** | **Yes** | **Yes** | **Yes** | No | No | No | No |
| 24† | 10 | 9 | 13 | 1 (0.29) / 2 (2.86) / 3 (1.03) / **4 (1.7) / 5 (0.36)** | -4 | **2** | **0** | **-2** | **1** | **0** | No | No | No | No | No | No | No |
| 25 | 11 | 9 | 10 | 1 (1.44) / 2 (2.24) / 3 (0.15) / **4 (1.0) / 5 (0.01)** | **-3** | **-4** | **-4** | -1 | **-2** | **-2** | No | No | No | No | No | No | No |
| 26† | 14 | 14 | 15 | 3 (0.51) | **1** | **1** | **1** | **0** | **0** | -1 | No | No | No | No | No | No | No |
| 27 | 2 | 2 | 5 | 1 (0.27) / 2 (1.93) / **5 (0.86)** | **-4** | **-5** | **-4** | **-2** | **-2** | **-2** | No | No | No | No | No | No | No |
| 28 | 16 | 9 | 2 | 1 (0.16) / 2 (1.42) / **4 (1) /** **5 (0.11)** | **0** | **-4** | **-5** | **0** | **-2** | **-3** | No | No | **Yes** | No | No | No | No |
| 29† | 4 | 2 | 2 | 1 (0.21) / 2 (2.29) / 3 (0.05) / **5 (1.16)** | **-5** | **-5** | **-4** | **-2** | **-3** | **-2** | **Yes** | **Yes** | **Yes** | No | No | No | No |
| 30 | 4 | 4 | 12 | 1 (0.16) / 2 (1.51) / 3 (0.33) / **5 (0.11)** | **-5** | **-4** | -2 | **-3** | **-2** | -1 | No | No | No | No | No | No | No |
| 32 | 10 | 8 | 8 | 1 (0.20) / 2 (2.43) / 3 (0.26) / **5 (0.63)** | **-3** | **-4** | **-4** | -1 | **-2** | **-2** | No | No | No | No | No | No | Yes |
| 33† | 5 | 5 | 2 | 1 (0.17) / 2 (2.18) / **5 (0.07)** | **-4** | **-4** | **-5** | **-3** | **-3** | **-3** | No | **Yes** | **Yes** | No | No | No | No |
| **EEG criterion missing, n = 7** | | | | | | | | | | | | | | | | | |
| 1 | 16 | 16 | 16 | None | **0** | **0** | **0** | **0** | **0** | **0** | No | No | No | No | No | No | - |
| 8 | 15 | 16 | 16 | None | **0** | **0** | **0** | **0** | **0** | **0** | No | No | No | No | No | No | - |
| 16 | 14 | 16 | 14 | None | **0** | **-1** | **0** | **-1** | **0** | **-1** | No | No | No | No | No | No | - |
| 18†# | 3 | 4 | 4 | 1 (0.19) / 2 (1.94) / **5 (0.05)** | **-5** | **-5** | **-5** | **-3** | **-3** | **-3** | **Yes** | **Yes** | **Yes** | No | No | No | - |
| 21 | 6 | 6 | 7 | 1 (0.26)/ 2 (2.69) | **-4** | **-4** | **-4** | **-3** | **-3** | **-3** | No | No | No | No | No | No | - |
| 22 | 2 | 1 | 3 | 1 (0.30) / 2 (3.36) / **5 (1.05)** | **-5** | **-5** | **-5** | **-3** | **-3** | **-3** | **Yes** | **Yes** | **Yes** | No | No | No | - |
| 31† | 0 | 2 | 2 | 1 (0.03) / 2 (3.40) / **5 (3.52)** | **-5** | **-5** | **-5** | **-3** | **-3** | **-3** | No | No | No | No | No | No | - |

FOUR - Full Outline of Unresponsiveness, RASS – Richmond Agitation Sedation Scale, SBS - State Behavioral Scale, EEG – Electroencephalogram.

^a^Hatched cells represent the presence of a diagnostic criterion. ^b^1 Midazolam (mg/kg/h); 2 Fentanyl (µg/Kg/h); 3 Dexmedetomidine (µg/kg/h);

4 Propofol (mg/kg, bolus, total per day); 5 Ketamine (mg/kg/h); ^c^Propofol and ketamine doses in bold; ^d^Not using propofol or ketamine on day 3.

†Patients with abnormal levels of NSE or S100B. # The only deceased patient.

**Table S5.** Other clinical characteristics: PELOD-2 score, CRP levels, need for vasopressor and/or inotropic drugs, neurological examination, functional status scale, and infectious agent.

|  | **PELOD-2** | | | | | | | **Median (IQR)** | **CRP (mg/dL)**  **Days of evolution** | | | **Hemody-namic support**  **(up to Dday)** | **Neurological examination** | | | | | | | **Functional Status** | | | | | **Isolated infectious agent** |
| --- | --- | --- | --- | --- | --- | --- | --- | --- | --- | --- | --- | --- | --- | --- | --- | --- | --- | --- | --- | --- | --- | --- | --- | --- | --- |
| **Patient*** | **Days of evolution** | | | | | | |  |  |  |  |  | **Days of evolution** | | | | | | | **Scale (FSS)** | | | | |  |
|  | **1** | **2** | **3** | **4** | **5** | **6** | **7** |  | **1** | **3** | **7** |  | **1** | **2** | **3** | **4** | **5** | **6** | **7** | **Adm.** | **Disc.** | **3m** | | **6m** |  |
| 2 | 1 | 3 | 3 | 0 | 0 | 0 | 0 | 0 (0-2) | 26,19 | 11,3 | 1,2 | D1^h^ | N | N | N | N | Ab^a^ | Ab^b^ | Ab^b^ | 6 | 6 | 6 | | 6 | *H. influenzae* |
| 3# | 1 | 0 | 0 | 0 | 0 | 0 | 0 | 0 (0-0) | - | - | - | D4^j^ | N | N | N | N | N | N | N | 6 | 6 | 6 | | 6 | - |
| 4 | 2 | 6 | 8 | 8 | 9 | 9 | 9 | 8 (7-9) | 21,25 | 19,75 | - | D7^h,i,j,k^ | Ab^g^ | Ab^c,d^ | **NMB** | **NMB** | Ab^c,d^ | Ab^b,c^ | Ab^b^ | 6 | 6 | 6 | | 6 | *Proteus sp.* |
| 5 | 3 | 4 | 3 | 4 | 3 | 3 | 3 | 3 (3-3.5) | 5,9 | 0,2 | - | D2^h^ | **NMB** | **NMB** | Ab^c,d,e,f^ | Ab^c,d,e^ | N | Ab^b^ | Ab^b^ | 6 | **7** | 6 | | 6 | - |
| 6# | 2 | 3 | 3 | 4 | 4 | 4 | 4 | 4 (3-4) | - | - | - | D7^h,k^ | Ab^b,c,d,e^ | Ab^b,c,d,e^ | N | N | N | Ab^a,c^ | Ab^b,c^ | 6 | 6 | 6 | | 6 | - |
| 7† | 1 | 3 | 3 | 4 | 4 | 4 | 5 | 4 (3-4) | 12,14 | 12,27 | - | D7^i,k^ | Ab^c,d^ | N | **NMB** | **NMB** | Ab^c,d^ | N | N | 6 | 6 | 6 | | 6 | *Adenovirus* |
| 9† | 1 | 5 | 3 | 4 | 3 | 4 | 4 | 4 (3-4) | - | - | - | D7^k^ | Ab^b,c^ | Ab^b,c,d,e^ | N | N | N | Ab^b^ | Ab^b^ | 6 | 6 | 6 | | 6 | *H. influenzae* |
| 10 | 2 | 3 | 3 | 4 | 4 | 4 | 4 | 4 (3-4) | 4,2 | 1,4 | 0,4 | D7^i^ | Ab^c,d^ | Ab^a,b,c,d^ | Ab^c^ | Ab^c^ | Ab^b,c^ | Ab^b,c,d^ | Ab^b,c,d^ | 6 | 6 | 6 | | 6 | - |
| 11 | 3 | 4 | 4 | 4 | 4 | 4 | 4 | 4 (4-4) | 29,7 | 20,6 | - | D7^h,i,k^ | N | Ab^b,d^ | Ab^b,d^ | Ab^b,c,d^ | Ab^b,c,d^ | Ab^b^ | Ab^b^ | 6 | **7** | **7** | | 6 | *H. influenzae* |
| 12 | 1 | 5 | 0 | 0 | 0 | 0 | 0 | 0 (0-0.5) | 30,5 | 14,7 | 10,7 | D2^i^ | N | N | N | N | N | N | N | 6 | 6 | 6 | | 6 | - |
| 13† | 0 | 0 | 0 | 0 | 0 | 0 | 0 | 0 (0-0) | 8,0 | 0,4 | 0,3 | - | N | N | N | N | N | N | N | 6 | 6 | 6 | | 6 | *E. coli* |
| 14# | 2 | 3 | 3 | 4 | 3 | 3 | 3 | 3 (3-3) | 30,0 | 16,5 | 8,6 | D3^i^ | Ab^b,c,d^ | Ab^b,c,d^ | Ab^b,c,d^ | Ab^b,d^ | Ab^b^ | Ab^c^ | N | 6 | 6 | 6 | | 6 | - |
| 15# | 1 | 3 | 3 | 0 | 0 | 0 | 0 | 0 (0-2) | 1,4 | 0,5 | - | D3^h,i^ | Ab^c,d^ | Ab^c,d^ | N | N | N | N | N | 6 | 6 | 6 | | 6 | *Influenza A* |
| 17† | 3 | 3 | 3 | 4 | 3 | 3 | 3 | 3 (3-3) | 5,1 | 2,6 | 0,5 | D7^i,j^ | N | N | Ab^c^ | Ab^d^ | Ab^b,c^ | Ab^b,c^ | N | 6 | 6 | 6 | | 6 | *Influenza B* |
| 19† | 2 | 3 | 4 | 5 | 5 | 5 | 5 | 5 (3.5-5) | 14,7 | 2,9 | 0,2 | D4^i^ | Ab^b,c,d^ | Ab^b,c,d^ | Ab^b,c^ | Ab^b,c^ | Ab^b,c,d^ | Ab^b,c^ | Ab^b,c^ | 6 | 6 | 6 | | 6 | *S. viridans* |
| 20 | 2 | 0 | 0 | 0 | 0 | 0 | 0 | 0 (0-0) | 20,6 | 16,1 | 8,4 | D7^i,j^ | N | N | N | N | N | N | N | 6 | 6 | 6 | | 6 | *S. pyogenes* |
| 23 | 4 | 7 | 6 | 6 | 6 | 6 | 6 | 6 (6-6) | 33,4 | 5,6 | 0,8 | D7^h,i^ | **NMB** | **NMB** | **NMB** | Ab^b,c,d^ | Ab^b,c,d^ | Ab^b,c,d^ | Ab^c^ | 6 | 6 | 6 | | 6 | *Influenza B* |
| 24† | 0 | 0 | 0 | 0 | 0 | 0 | 0 | 0 (0-0) | 5,5 | 4,3 | 1,3 | D6^h,i^ | N | N | N | N | N | N | N | 6 | 6 | 6 | | 6 | - |
| 25 | 0 | 0 | 0 | 1 | 1 | 1 | 1 | 1 (0-1) | 4,5 | 1,2 | < 0,03 | D7^h,i^ | Ab^c,d^ | Ab^c,d^ | Ab^c,d^ | N | N | N | N | 6 | 6 | 6 | | 6 | *S. epidermidis* |
| 26† | 1 | 0 | 0 | 0 | 0 | 0 | 0 | 0 (0-0) | 31,1 | 7,4 | 0,9 | D4^i^ | N | N | N | N | N | N | N | 6 | 6 | 6 | | 6 | *Influenza A* |
| 27 | 0 | 3 | 3 | 4 | 3 | 3 | 3 | 3 (3-3) | 7,1 | 2,4 | 0,5 | D7^i^ | Ab^b,c,d^ | Ab^c,d^ | Ab^c,d^ | Ab^c,d^ | N | N | N | 6 | 6 | 6 | | 6 | *P. aeruginosa* |
| 28 | 0 | 3 | 3 | 4 | 4 | 4 | 4 | 4 (3-4) | 1,3 | 2,8 | 2,3 | D7^h,i^ | N | Ab^b,c^ | **NMB** | **NMB** | **NMB** | **NMB** | **NMB** | 6 | 6 | 6 | | 6 | *Influenza B* |
| 29† | 0 | 4 | 6 | 7 | 7 | 7 | 7 | 7 (5-7) | 2,6 | 1,8 | < 0,03 | D7^i^ | **NMB** | **NMB** | **NMB** | **NMB** | **NMB** | **NMB** | **NMB** | 6 | 6 | 6 | | 6 | - |
| 30 | 2 | 4 | 4 | 3 | 0 | 0 | 0 | 2 (0-3.5) | 45,2 | 4,9 | < 0,03 | D4^h^ | Ab^c,d^ | Ab^c,d^ | N | N | N | N | N | 6 | 6 | 6 | | 6 | - |
| 32# | 0 | 3 | 3 | 4 | 4 | 4 | 3 | 3 (3-4) | 33,4 | 22,7 | - | D7^h,i^ | N | Ab^c,d^ | Ab^c,d^ | **NMB** | **NMB** | Ab^c,d^ | Ab^c,d^ | 6 | 6 | 6 | | 6 | - |
| 33† | 2 | 6 | 5 | 6 | 6 | 6 | 5 | 6 (5-6) | 34,4 | 13,1 | 3,4 | D7^i^ | Ab^c,d^ | **NMB** | **NMB** | **NMB** | **NMB** | N | N | 6 | 6 | 6 | | 6 | - |
| **EEG criterion missing, n = 7** | | | | | | | | | | | | | | | | | | | | | | |  |  |  |
| 1 | 1 | 3 | 0 | 0 | 0 | 0 | 0 | 0 (0-5) | - | - | - | D1^h^ | N | N | N | N | N | N | N | 6 | 6 | 6 | | 6 | *S. epidermidis* |
| 8 | 2 | 0 | 0 | 0 | 0 | 0 | 0 | 0 (0-0) | - | - | - | D1^k^ | Ab^g^ | N | N | N | N | N | N | 6 | 6 | 6 | | 6 | *S. aureus MRSA* |
| 16 | 0 | 0 | 0 | 0 | 0 | 0 | 0 | 0 (0-0) | 47,4 | 16,3 | - | - | Ab^b,c,d^ | N | Ab^b^ | Ab^b,g^ | N | N | N | 6 | **7** | 6 | | 6 | - |
| 18† | 1 | 8 | 6 | 7 | 7 | 7 | 7 | 7 (6.5-7) | 20,7 | 26,9 | 1,1 | D7^h,i,j,l^ | **NMB** | **NMB** | **NMB** | **NMB** | **NMB** | **NMB** | **NMB** | 6 | **D** | **-** | | **-** | **-** |
| 21 | 4 | 4 | 3 | 4 | 3 | 3 | 3 | 3 (3-4) | 38,3 | 6,9 | 0,7 | D7^h,j^ | Ab^c,d^ | Ab^b,c,d^ | Ab^b,c,d^ | Ab^b,c,d^ | Ab^b,c^ | N | N | 6 | 6 | 6 | | 6 | *E. coli* |
| 22 | 2 | 4 | 4 | 5 | 5 | 5 | 2 | 4 (3-5) | 6,1 | 2,8 | 1,3 | D7^j^ | **NMB** | **NMB** | **NMB** | **NMB** | Ab^b,c^ | Ab^b,c^ | Ab^b,c^ | 6 | 6 | 6 | | 6 | *Acinctobacter sp.* |
| 31† | 4 | 8 | 13 | 10 | 9 | 9 | 9 | 9 (8.5-9.5) | 16,5 | 25,2 | 34,9 | D7^h,i,m^ | Ab^c,d^ | Ab^c,d^ | Ab^c,d^ | Ab^c,d^ | Ab^c,d^ | Ab^c,d^ | Ab^c,d^ | 6 | 6 | 6 | | 6 | *S. pyogenes* |

PELOD-2 – Pediatric Logistic Dysfunction 2, CRP – C-reactive protein, EEG – electroencephalogram, IQR – Interquartile Range, N – Normal, Ab – Abnormal, NMB – Neuromuscular blocking, D - Deceased, PICU – Pediatric Intensive Care Unit, Adm. – Admission, Disc. – Discharge, 3m and 6 m– three and six months after hospital discharge, LOS – length of stay. a – tremor, b – hypoactivity, c – miosis, d - absence of eye contact, e - absent corneal-eyelid reflex, f - absent plantar reflex, g – abnormal respiratory pattern, h – noradrenaline, i – adrenaline, j – milrinone, k – dobutamine, l – nitroprusside, m – vasopressin.

*Hatched cells represent a presumptive diagnosis of sepsis-associated encephalopathy. #Patients with EEG suggestive of acute encephalopathy. †Patients with abnormal levels of NSE or S100B. ∆ Patient with no clinical findings suggestive of SAE but missing the EEG.

**Table S6.** Electroencephalographic characteristics of 26 patients who had the opportunity to undergo this exam (continue next page).

| **Patient**  **No.** | **Day** | **Sedative drugs and doses^a^** | **RASS** | **NMB** | **Anti-convulsant** | **EEG duration (minutes)** | **Graphoelements of sleep^b^** | **Continuous slowing** | **Intermittent slowing** | **Predominant rhythm** | **Interictal epileptiform discharges** | **TW** |
| --- | --- | --- | --- | --- | --- | --- | --- | --- | --- | --- | --- | --- |
| 2 | D1 | 1 (0.3) / 2 (3.1) /  3 (1) / 4 (4.8) / 5 (0.2) | -3 | No | Phenytoin | 720 | 1, 2, 3 | Yes, diffuse | No | Delta | No | No |
| 3 | D6 | None | 0 | No | No | 37 | N/A, awake | Yes, diffuse | No | Theta | No | No |
| 4 | D3 | 1 (0.3) / 2 (2.9) | -5 | Yes | No | 720 | 1, 2, 3 | Yes, diffuse | No | Delta | No | No |
| 5 | D3 | 1 (0.2) / 2 (2)  3 (0.9) / 5 (0.3) | -5 | No | No | 360 | 1, 2, 3 | Yes, diffuse | No | Delta/Beta | No | No |
| 6 | D7 | 1 (0.3) / 2 (3.2)  3 (0.5) / 5 (0.05) | -4 | No | No | 720 | 1, 2, 3 | Yes, diffuse | Yes, parietal, occipital, right | Delta | Yes, frontal, parietal, occipital, right | No |
| 7 | D3 | 1 (0.2) / 2 (2) | -5 | No | No | 720 | 1, 3 | Yes, diffuse | No | Delta/Beta | No | No |
| 9 | D5 | 1 (0.3) /2 (2) /3 (0.9) 4 (4.8) | -2 | No | No | 600 | Spindles | Yes, diffuse | No | Delta/Theta | No | No |
| 10 | D4 | 1 (0.1) / 2 (0.7)  3 (0.3) | -3 | No | No | 64 | Absent | Yes, diffuse | No | Delta/Beta | No | No |
| 11 | D3 | 1 (0.06) / 2 (0.3)  3 (0.3)/ 5 (0.05) | -4 | No | No | 615 | Absent | Yes, diffuse | No | Delta | No | No |
| 12 | D4 | 3 (0.1) | 1 | No | No | 70 | Absent | Yes, diffuse | Yes, central and parietal, right | Delta/Theta | Yes, central, parietal | No |
| 13 | D2 | None | 0 | No | No | 422 | Spindles, bilateral | Yes, diffuse | No | Delta/Theta | No | No |
| 14 | D6 | 1 (0.06) / 2 (0.6)  3 (0.09) | -1 | No | No | 600 | Spindles, bilateral | Yes, diffuse | No | Delta/Theta | Yes, frontal | No |
| 15 | D3 | 1 (0.07)  2 (0.78) / 3 (0.6) | -3 | No | Phenobarbital, phenytoin | 364 | Spindles, bilateral | Yes, diffuse | Yes, temporal, right | Delta/Theta | No | No |
| 17 | D2 | 1 (0.3) / 2 (3)  5 (0.5) | -4 | No | No | 55 | Absent | Yes, diffuse | No | Delta/Theta | No | No |
| 19 | D2 | 1 (0.6) / 2 (5.3)  5 (0.3) | -3 | No | No | 83 | Absent | Yes, diffuse | No | Delta/Theta | No | No |

RASS – Richmond Agitation Sedation Scale, NMB – Neuromuscular block, EEG – electroencephalogram, TW – Triphasic waves, N/A – not applicable

^a^ 1 Midazolam (mg/kg/h); 2 Fentanyl (µg/kg/h); 3 Dexmedetomidine (µg/Kg/h); 4 Propofol (mg/kg, bolus, total per day); 5 Ketamine (mg/kg/h)

^b^ 1 Spindles, 2 Vertex sharp-waves, 3 K-Complex

**Table S6 (continued from the previous page).** Electroencephalographic characteristics of 26 patients who had the opportunity to undergo this exam.

| **Patient**  **No.** | **Day** | **Sedative drugs and doses^a^** | **RASS** | **NMB** | **Anti-convulsant** | **EEG duration (minutes)** | **Graphoelements of sleep^b^** | **Continuous slowing** | **Intermittent slowing** | **Predominant rhythm** | **Interictal epileptiform discharges** | **TW** |
| --- | --- | --- | --- | --- | --- | --- | --- | --- | --- | --- | --- | --- |
| 20 | D2 | None | -1 | No | No | 708 | Spindles, bilateral | No | No | Beta when awake, Delta in sleeping | No | No |
| 23 | D5 | 1 (0.2) / 2 (2.6)  5 (1.7) | -5 | No | No | 396 | Absent | Yes, diffuse | No | Delta | No | No |
| 24 | D7 | 1 (0.3) / 2 (0.4)  3 (1.1) / 5 (1) | -2 | No | No | 518 | Absent | Yes, diffuse | Yes, diffuse | Delta | No | No |
| 25 | D4 | 1 (0.3) / 2 (4)  3 (1.6) / 5 (0.9) | -3 | No | No | 534 | Absent | Yes, diffuse | No | Delta | No | No |
| 26 | D4 | 3 (0.5) | 1 | No | No | 634 | Absent | Yes, diffuse | No | Delta/Theta | No | No |
| 27 | D3 | 1 (0.3) / 2 (2)  5 (0.8) | -4 | No | No | 198 | Absent | Yes, diffuse | No | Delta | No | No |
| 28 | D7 | 1 (0.2) / 2 (2) / 5 (0.6) | -5 | Yes | No | 653 | Absent | Yes, diffuse | No | Delta | No | No |
| 29 | D3 | 1 (0.3) / 2 (3.4)  3 (0.2) / 5 (1.2) | -4 | Yes | No | 208 | Absent | Yes, diffuse | No | Delta/Theta | No | No |
| 30 | D4 | 1 (0.2) / 2 (1.9)  3 (0.5) | -2 | No | No | 115 | Absent | Yes, diffuse | No | Delta/Theta | No | No |
| 32 | D4 | 1 (0.3) / 2 (3.1)  3 (0.14) / 5 (0.27) | -5 | Yes | No | 117 | Spindles, right | Yes, diffuse | No | Delta/Theta | Yes, frontal, left | No |
| 33 | D6 | 1 (0.2) / 2 (2.9) | 0 | No | No | 241 | Absent | No | Yes, diffuse | Theta | No | No |

RASS – Richmond Agitation Sedation Scale, NMB – Neuromuscular block, EEG – electroencephalogram, TW – Triphasic waves

^a^ 1 Midazolam (mg/kg/h); 2 Fentanyl (µg/kg/h); 3 Dexmedetomidine (µg/kg/h); 4 Propofol (mg/kg, bolus, total per day); 5 Ketamine (mg/kg/h)

^b^ 1 Spindles, 2 Vertex sharp-waves, 3 K-Complex
